# From Structure to Function: 2-Chloro-5-nitrobenzoic Acid Derivatives as Potential Next-Generation Antibacterials

**DOI:** 10.3390/ijms262311607

**Published:** 2025-11-29

**Authors:** Lilia Croitor, Anastasia Gorobet, Marioara Nicoleta Caraba, Pavlina Bourosh, Ion Valeriu Caraba, Daniela Haidu, Manuela Crisan

**Affiliations:** 1 Institute of Applied Physics, Moldova State University, 5 Academiei Str., MD2028 Chisinau, Moldova; lilia@gmail.com (L.C.); nasteagorobet@gmail.com (A.G.); bourosh.xray@gmail.com (P.B.); 2 Department of Cellular and Molecular Biology, Faculty of Medicine, “Victor Babeș” University of Medicine and Pharmacy Timisoara, 300041 Timisoara, Romania; nicoleta.caraba@umft.ro; 3 ANAPATMOL Research Center, “Victor Babeș” University of Medicine and Pharmacy Timisoara, 300041 Timisoara, Romania; caraba_i@animalsci-tm.ro; 4 Faculty of Bioengineering of Animal Resources, University of Life Sciences “King Mihai I” from Timisoara, 119 Calea Aradului, 300645 Timisoara, Romania; 5 “Coriolan Dragulescu” Institute of Chemistry, Romanian Academy, 24 Mihai Viteazu Bd., 300223 Timisoara, Romania; dana_141@yahoo.com

**Keywords:** drug-resistant bacteria, bioactive molecules, chloro-nitro derivatives, alkanolamine, organic multicomponent system, coordination polymer, antibacterial compounds

## Abstract

The rapid emergence of drug-resistant bacteria demands alternative antimicrobial strategies that extend beyond conventional drugs. In this study, we present the synthesis, structural characterization, and antibacterial evaluation of two new 2-chloro-5-nitrobenzoic acid (2Cl5NBH) derivatives: a methylethanolammonium salt (compound **1**) and a 2D coordination polymer (compound **2**). Structural characterization by single-crystal X-ray diffraction, complemented by Hirshfeld surface analysis, revealed the supramolecular architectures and highlighted the key intermolecular interactions, providing essential insights into the potential role of these compounds in modulating their physicochemical and biological behavior. Antibacterial assays demonstrated that compound **1** exhibited a broad inhibitory profile against both Gram-positive and Gram-negative bacteria. In contrast, compound **2** exhibited selective inhibition against methicillin-resistant *Staphylococcus aureus* (MRSA) comparable to that of gentamicin.

## 1. Introduction

The rise of bacterial resistance to commonly used antibiotics represents one of the most serious challenges in contemporary medicine. The widespread and often uncontrolled use of antibiotics in healthcare and agriculture has accelerated the emergence and spread of multidrug-resistant bacterial strains [1]. This limited the efficacy of conventional drugs, contributing to increased mortality rates, more extended hospitalizations, and higher healthcare costs [2,3]. Bacterial species such as *Staphylococcus aureus*, *Salmonella typhimurium*, and *Escherichia coli* are now commonly encountered in both clinical and community settings, and many isolates exhibit high levels of resistance [4,5,6]. Meanwhile, the slow progress in developing new antibiotics highlights the urgent need for alternative antibacterial agents that act through new or complementary mechanisms to existing treatments.

Among the promising molecular scaffolds, aromatic carboxylic acids, particularly benzoic acid derivatives, have attracted scientific interest due to their antimicrobial potential, structural versatility, chemical stability, and relatively low toxicity [7,8]. Their longstanding use as food preservatives also supports their potential for safe therapeutic applications [9]. Improving the efficacy of antibacterial compounds, especially against resistant bacterial strains, requires structural modifications to enhance membrane bacterial permeability, target affinity, or intracellular retention. Key molecular parameters, such as polarity, lipophilicity, or non-covalent interaction capacity, can be influenced by substitution with electron-withdrawing groups (EWGs), the formation of multicomponent crystalline systems (such as salts and cocrystals), or coordination with metal ions [10,11,12,13,14]. The introduction of EWGs, for example, nitro (–NO_2_) and halogen (–Cl) substituents, can modulate the electron density within the molecule, influencing the compound’s reactivity, intermolecular interactions, and membrane permeability.

In addition to electronic effects, multicomponent crystalline systems provide an alternative strategy for modulating biological activity. Salt formation can enhance several physicochemical properties, such as aqueous solubility, bioavailability, and stability, which play crucial roles in the interaction of a compound with bacterial targets [15,16,17,18]. Alkanolamines, in particular, represent attractive counterions due to their favorable biocompatibility and structural similarity to ethanolamine, an essential component of membrane phospholipids [19,20]. Although direct experimental proof that ethanolamine-like counterions enhance membrane affinity or antibacterial activity remains limited, their chemical flexibility and hydrophilicity make them promising counterions for improving solubility and optimizing biological performance. 

Moreover, structural membrane differences between Gram-positive and Gram-negative bacteria influence the mechanism of action of these compounds. In Gram-negative bacteria, the presence of an outer lipopolysaccharide (LPS) membrane and porin channels can limit permeability. Still, compounds with improved solubility and ionization profiles may facilitate transmembrane diffusion and reduce active efflux. In contrast, Gram-positive bacteria, characterized by a thick, negatively charged peptidoglycan layer and the absence of an outer membrane, may be more susceptible to electrostatic interactions that disrupt membrane structure or inhibit enzymatic activity.

Metal complexation can enhance lipophilicity, stability, and cellular uptake through novel mechanisms of action [21,22]. Transition metals such as Cu^2+^ and Fe^2+^/Fe^3+^ often act through redox cycling, generating reactive oxygen species (ROS) that induce oxidative stress and damage bacterial membranes, proteins, and DNA. Other metals, including Zn^2+^, Ag^+^, Co^2+^, and Ni^2+^, act via non-redox pathways such as enzyme inhibition or disruption of nucleic acid function [23,24]. In contrast, potassium, although essential for bacterial homeostasis and signaling, remains relatively underexplored in this context. Given its role in osmoregulation, pH balance, membrane potential, and protein synthesis [25], K^+^-based complexes became attractive for exploring alternative antimicrobial strategies.

Based on these considerations, the present study reports the synthesis, structural characterization, and antibacterial evaluation of two new derivatives of 2-chloro-5-nitrobenzoic acid (2Cl5NBH): a methylethanolammonium salt and a potassium coordination complex. In addition to crystallographic analysis, Hirshfeld surface mapping and 2D fingerprint analysis were employed to gain deeper insight into the intermolecular interactions of supramolecular architectures and to correlate structural features with observed antibacterial activity. The antibacterial potential was assessed in comparison with the parent 2Cl5NBH and standard antibiotics against representative Gram-positive and Gram-negative bacterial strains, including clinical isolates and methicillin-resistant *Staphylococcus aureus* (MRSA). Antibacterial efficacy was systematically evaluated through inhibition zone measurement, bacterial cell viability assays, and biofilm inhibitory tests. Furthermore, based on the structural data and relevant literature, plausible modes of interaction of the new compounds with bacterial cells are proposed, providing a foundation for future experimental investigations.

## 2. Results

### 2.1. Synthesis and Structural Characterization

**Scheme 1:**
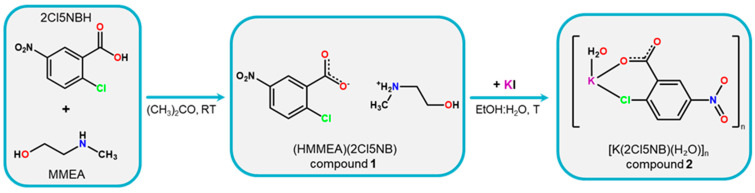
General method for preparation of compounds **1** and **2**.

The synthetic approach was designed to improve the physicochemical profile and supramolecular organization of 2Cl5NBH through the formation of two distinct derivatives: a methylethanolammonium salt (HMMEA)(2Cl5NB) (compound **1**) and the potassium coordination polymer [K(2Cl5NB)(H_2_O)]_n_ (compound **2**) (Scheme 1).

The molecular and supramolecular structures of both compounds were established by single-crystal X-ray diffraction (SCXRD) analyses. The crystallographic details of the structure refinement of compounds **1** and **2** are presented in Table 1, while Tables S1 and S2 feature selected geometric parameters.

**Figure 1:**
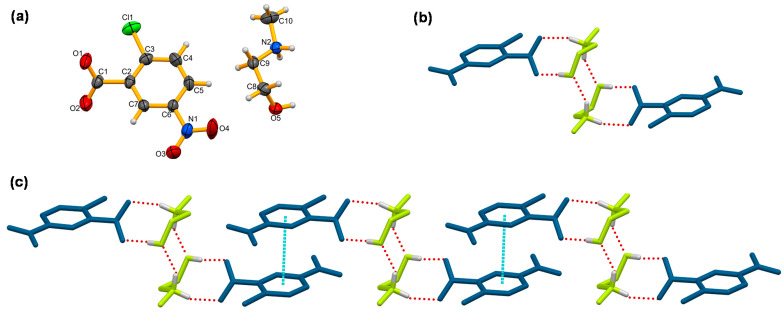
(**a**) The asymmetric unit of compound **1** with atomic numbering scheme. Thermal ellipsoids are depicted at a 50% probability level. (**b**) Hydrogen-bonded supramolecular centrosymmetric tetramer. (**c**) 1D supramolecular chain sustained by π–π interactions.

SCXRD analysis revealed that compound **1** crystallizes in the triclinic centrosymmetric space group *P*-1, with one cationic HMMEA^+^ and one anionic 2Cl5NB^−^ species in the asymmetric unit (Figure 1a). The supramolecular arrangement is formed via a proton transfer from the carboxylic acid group of 2Cl5NBH to the nitrogen atom of MMEA. The components are interconnected through two charge-assisted hydrogen bonds: O–H⋯O^−^ and N^+^–H⋯O. These interactions generate two R_2_^2^(9) heterosynthons and one R_2_^2^(10) homosynthon, leading to the formation of a supramolecular centrosymmetric tetramer (Figure 1b). The [(HMMEA)(2Cl5NB)]_2_ units organize into supramolecular 1D chains (Figure 1c) by π–π stacking interactions between adjacent phenyl rings of 2Cl5NB^−^ anions (centroid⋯centroid and plan⋯plan distances are 3.844 and 3.511 Å, respectively).

**Figure 2:**
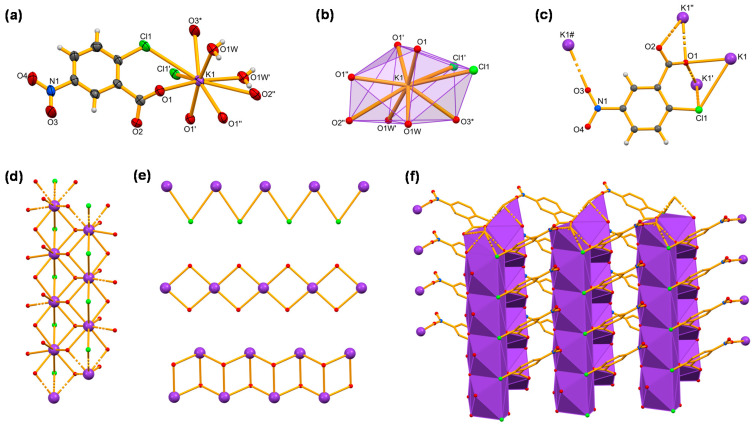
(**a**) Coordination environments of K(I) ions in compound **2** represented with thermal ellipsoids at a 50% probability level. (**b**) Schematic representation of the distorted KO_7_Cl_2_ tricapped trigonal prismatic coordination polyhedron in compound **2**. (**c**) Coordination modes of the 2Cl5NB^−^ ligand, along with symmetry transformation used to generate equivalent atoms in compound **2**: ′ = 1 + x, y, z; ″ = ½ + x, ½ − y, −z; * = 1 + x, −1 + y, z; # = −1 + x, −1 + y, z. (**d**) The ribbon-like chain [KO_7_Cl_2_]_n_ in **2**. (**e**) One zig-zag and two types of rhombohedral chains fused into the ribbon-like rod. (**f**) A sheet of rods in **2** formed by edge-sharing K^+^ polyhedra.

Building on our previous work, which demonstrated the formation of single-crystal complexes from organic multicomponent systems and metal ions [26,27,28], we extended the study to investigate the coordination behavior of compound **1** with K^+^ ions. This direction was motivated by the biological relevance, low toxicity, and well-documented ability of potassium to stabilize carboxylate-based coordination polymers. As a result was obtained compound **2**, a 2D potassium coordination polymer, [K(2Cl5NB)(H_2_O)]_n_, which crystallized as colorless single crystals. The absence of the methylethanolamine (MMEA) co-former in the final structure suggests a complete structural reorganization during coordination with K^+^, highlighting the system’s sensitivity to metal-mediated assembly. Compound **2** crystallizes in the orthorhombic non-centrosymmetric *P*2_1_2_1_2_1_ space group and features a layered polymeric structure formed by K(H_2_O)^+^ cations linked by 2Cl5NB^−^ anions. The asymmetric unit contains one crystallographically independent K^+^ ion, one 2Cl5NB^−^ anion, and one coordinated water molecule. Each K^+^ ion is nine-coordinated and adopts a distorted tricapped trigonal prismatic geometry (Figure 2a,b), defined by a O_7_Cl_2_ donor set. Five oxygen atoms and two chlorine atoms originate from four distinct 2Cl5NB^−^ anions, while two coordinated water molecules provide the other two oxygen atoms. The 2Cl5NB^−^ anion acts as a μ_4_-bridging, heptadentate ligand (Figure 2c), linking four K^+^ ions. It forms two bidentate chelations with two K^+^ ions through carboxylate oxygen and a chlorine atom (O1 and Cl1). Additionally, it forms another bidentate chelation with a third potassium ion, involving both oxygen atoms of the carboxyl group. Furthermore, it bridges a fourth metal ion via one oxygen atom (O3) of the nitro group, while O4 remains uncoordinated.

The symmetry-related KO_7_Cl_2_ polyhedra share common edges through bridging water molecules (O1W), as well as μ_2_-Cl1 and μ_3_-O1 atoms from the 2Cl5NB^−^ ligand. These polyhedra extend into ribbon-like rods (Figure 2d), formed by the fusion of three distinct types of chains (Figure 2e). The 2Cl5NB^−^ ligands further connect these rods into sheet-like structures with rhombic windows through the oxygen atoms of the nitro groups located on the opposite side of the ligand (Figure 2f). The excellent solubility of compound **2** in both water and DMSO supports its suitability for biological assays and pharmaceutical development.

Compound **1** exhibited a sharp melting transition at 91.2–91.3 °C (Figure S1a), indicating high purity and a well-ordered crystalline structure. In contrast, compound **2** did not melt up to 313 °C, but underwent gradual darkening and carbonization, indicating thermal decomposition (Figure S1b). This behavior correlates with its extended coordination network, involving K–O and K–Cl bonds, reinforced by hydrogen bonding and van der Waals interactions, which form a rigid crystalline structure.

**Figure 3:**
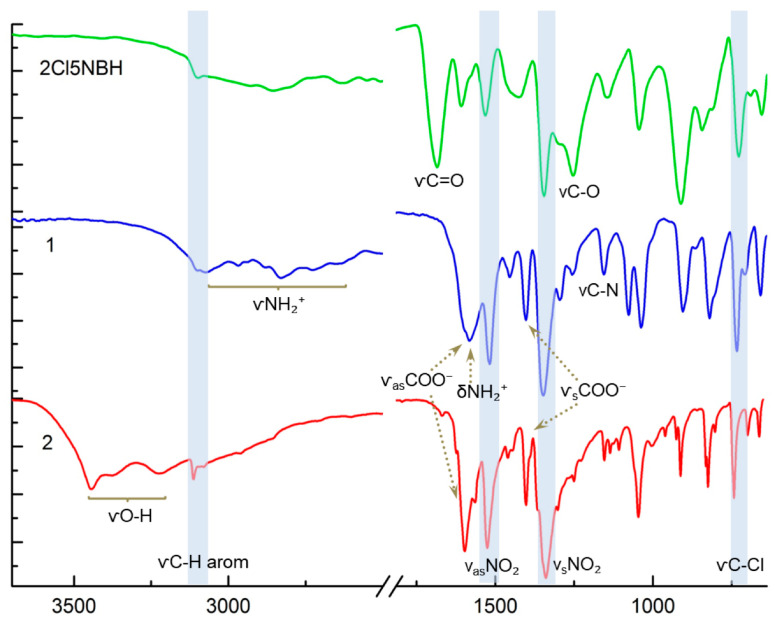
Comparable IR spectra for compounds **1**, **2** and 2Cl5NBH.

The IR spectrum of compound **1** (Figure 3) confirms salt formation via proton transfer and electrostatic interaction between the carboxylate anion and the MMEA cation. Compared to the parent acid 2Cl5NBH, several key spectral changes are observed: disappearance of νC=O stretching band (1700–1680 cm^−1^), characteristic of free –COOH group; appearance of νCOO^−^ asymmetric (1650–1540 cm^−1^) and symmetric (1450–1360 cm^−1^) stretching bands, indicating deprotonation and formation of the carboxylate anion; and a broad band around 1584 cm^−1^, resulting from the overlap of ν_as_COO^−^ with δNH_2_^+^ bending vibration, supporting the presence of the protonated amine. In the 1220–1050 cm^−1^ region, additional spectral changes confirm the structural transformation: disappearance of νC-O stretching band at 1250 cm^−1^ associated with –COOH group and emergence of νC-N band at 1076 cm^−1^ assigned to MMEA cation. Moreover, the presence of broad absorptions in 3100–2700 cm^−1^ and 2700–2300 cm^−1^ regions is attributed to the asymmetric and symmetric stretching vibrations of the protonated –NH_2_^+^ group, confirming the ionic nature of the compound [29,30].

For compound **2**, the spectrum indicates the presence of COO^−^ groups, capable of forming interactions with predominantly electrostatic character with the K^+^ ions, as well as the presence of coordinated H_2_O molecules. Slight shifts in the νCOO^−^ asymmetric and symmetric stretching bands, compared to compound **1**, suggest coordination with K^+^, in agreement with SCXRD data. Additionally, a broad νO-H (3400–3200 cm^−1^) stretching band confirms the presence of crystallization water. In all studied compounds, the presence of –NO_2_ and –Cl substituents on the aromatic ring is confirmed by the ν_as_NO_2_ (1550–1540 cm^−1^) and ν_s_NO_2_ (1355–1315 cm^−1^) bands, as well as by the νC-Cl (850–700 cm^−1^) stretching bands. The aromatic νC-H (3100–3000 cm^−1^) stretching bands appear without significant shifts, indicating the preservation of the aromatic core in all structures. A detailed correspondence between observed IR bands and their vibrational assignments is provided in Supplementary Materials (Table S3).

### 2.2. Computational Study

**Figure 4:**
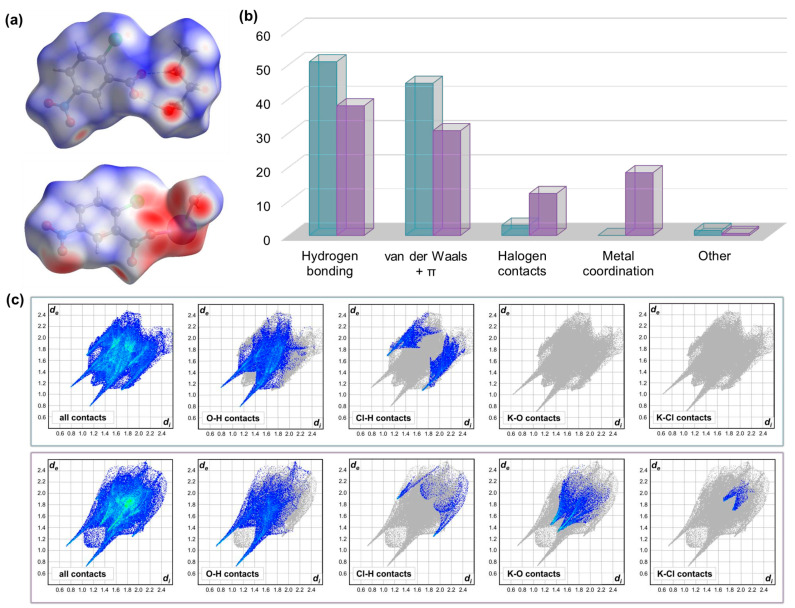
Hirshfeld surfaces (**a**), stacked bar chart of the relative contributions of major intermolecular interactions (**b**), and selected 2D fingerprint plots (**c**) in compounds **1** (**top**) and **2** (**bottom**).

To complement the crystallographic analysis and gain deeper insight into the intermolecular interactions governing the supramolecular architectures of compounds **1** and **2**. Hirshfeld surface analysis was conducted. The relative contributions of the principal interaction types are presented in Figure 4, while complete numerical data are available in the Supplementary Materials (Table S4). To avoid redundancy and ensure an unambiguous comparison, duplicate contacts contributing to multiple categories were excluded from the analysis.

Hydrogen bonding constitutes the dominant class of intermolecular interactions in both crystal structures, accounting 50.9% of the total surface contacts in compound **1** and 38.0% in compound **2**. The reduced contribution in compound **2** indicates the competition between coordination of oxygen donor atoms to the potassium center and classical O–H⋯O and Cl–H⋯O hydrogen bonds, thereby reducing the overall contribution of hydrogen bonding to crystal packing stabilization.

Metal coordination interactions, present exclusively in compound **2** (18.4%) involve K⋯O, K⋯Cl, and K⋯C linkages, confirming that the potassium cations function as structure-directing centers. Halogen contacts increase significantly from 3.0% in compound **1** to 12.3% in compound **2**, indicating enhanced participation of chlorine atoms in supramolecular stabilization through Cl⋯O and K⋯Cl linkages. Van der Waals and π-type interactions contribute 44.6% in compound **1** and 30.7% in compound **2**. The reduction in these weaker contacts upon coordination suggests a denser and more directional packing arrangement, wherein coordination bonds and hydrogen bonding predominate over dispersive interactions. A minor fraction of other contacts (1.5% and 0.6% for compounds **1** and **2**, respectively) completes the interaction profile.

From a surface polarity perspective, the balance between polar (hydrophilic) and nonpolar (lipophilic) interactions further illustrates this structural transformation. In compound **1**, hydrophilic contacts (hydrogen bonding and halogen contacts) account for approximately 53.9% of the total surface area, whereas lipophilic (van der Waals and π-type interactions) contribute 44.6%. In contrast, compound **2** exhibits a higher proportion of polar contacts (68.7%) due to the emergence of coordination linkages (K⋯O, K⋯Cl), with a corresponding decrease in nonpolar contributions (30.7%). The predominance of hydrophilic surface regions in compound **1**, associated with extensive O–H⋯O and Cl–H⋯O hydrogen bonding, suggests a stronger potential for interactions with polar molecules, including aqueous media and antibacterial agents. In contrast, the surface polarity of compound **2** is more localized around the potassium coordination nodes, producing a less homogeneous hydrophilic profile. These differences in the nature and proportion of interactions may influence the physicochemical behaviour and potential interactions with biological targets.

### 2.3. Antibacterial Activity

**Figure 5:**
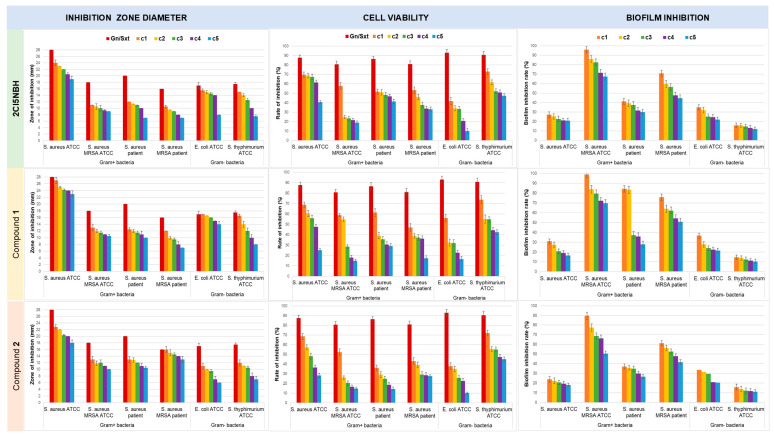
Evaluation of inhibition zone diameters (mm), rate of inhibition (%), and biofilm inhibition rate (%) induced by the tested compounds against Gram-positive and Gram-negative bacteria. Colors indicate compound concentrations (c1–c5). Gn and Sxt were used as reference antibiotics for Gram-positive and Gram-negative bacteria, respectively.

The antibacterial potential of both compounds and their corresponding acid, 2Cl5NBH, was assessed against six bacterial strains (four Gram-positive and two Gram-negative), including both reference strains (ATCC) and clinical isolates, as well as antibiotic-resistant variants. The assays were conducted under aqueous conditions (phosphate-buffered saline-PBS, pH 7.4) using standardized methods: zone of inhibition diameter (mm), bacterial cell viability (%), and biofilm inhibition (%) (Figure 5). Considering the predominance of H-bonding and weak van der Waals/π-type interactions observed in the crystal structures, both compounds are expected to dissociate in PBS, leading to ionic species as the predominant forms in solution. In the case of compound **2**, small aggregates may also persist, stabilized by van der Waals or H-bonding interactions. Consequently, the antibacterial activity observed is attributed mainly to these ionic species. For textual coherence, we continue to refer to the tested compounds as compound **1** and compound **2**, as named in the previous sections.

The disk diffusion test revealed strain-dependent differences in antibacterial activity among the tested compounds. Among Gram-positive bacteria, *S. aureus* ATCC was the most responsive. Compound **1** exhibited the most potent antibacterial activity, with inhibition zones against *S. aureus* ATCC comparable to gentamicin (Gn, 28 mm) at the highest concentration (c1, 27 mm) and gradually decreasing to 23 mm at c5, indicating an apparent dose-dependent effect. The parent acid 2Cl5NBH and compound **2** followed closely, with inhibition zones of 24–19 mm and 23–18 mm, respectively, also showing concentration-dependent responses. For the MRSA clinical isolate, compound **2** showed inhibition zones comparable to Gn (16 mm) at c1, with a slight reduction to 13 mm at c5. This behavior suggests a relevant inhibitory effect against resistant *S. aureus*. In contrast, compounds **1** and 2Cl5NBH showed moderate activity against MRSA under similar conditions.

Gram-negative strains (*E. coli* ATCC and *S. typhimurium* ATCC) exhibited a lower sensitivity to the tested compounds compared with *S. aureus* ATCC, probably due to the presence of the outer membrane rich in lipopolysaccharides. Compound **1** produced the largest inhibition zones (17–14 mm across c1–c5), comparable at higher concentrations to those obtained for sulfamethoxazole-trimethoprim (Sxt, 17 mm), particularly against *E. coli*. The parent acid 2Cl5NBH showed moderate activity (15–14 mm at c1–c4), while compound **2** exerted weak inhibition (<12 mm) over the same concentration range.

The bacterial cell viability assay, which quantifies bactericidal efficacy through inhibition rates, confirmed a clear concentration-dependent response in all tested strains. Compound **1** demonstrated superior antibacterial activity compared to compound **2**, especially against *S. aureus* ATCC and *E. coli*/*S. thyphimurium* ATCC at higher concentrations. These results support the hypothesis that both ionic components of salt can contribute to improved physicochemical behavior and antibacterial activity by a synergistic effect. In contrast, compound **2** showed limited inhibition of bacterial viability, particularly in *S. aureus* clinical isolates, due to reduced intracellular accumulation or weaker ability to interfere with essential bacterial targets. The 2Cl5NBH produced moderate viability inhibition in Gram-positive strains, consistent with the inhibition zones obtained in the disk diffusion test.

The biofilm inhibition potential of the synthesized compounds was assessed to evaluate their ability to prevent biofilm formation, a critical factor in the persistence of chronic and antibiotic-resistant infections. The results showed that all three compounds reduced biofilm formation in a concentration-dependent manner, with maximum inhibition at c1 and progressively reduced activity from c2 to c5. This trend reflects the results of disk diffusion and cell viability assays, particularly for Gram-negative strains, highlighting the relationship between antibacterial activity and biofilm inhibition.

Overall, biofilm inhibition was more pronounced in Gram-positive strains than in Gram-negative ones. This difference can be attributed to structural differences in their cellular membrane structure. Notably, the results of the biofilm inhibition assay revealed a markedly enhanced effect in antibiotic-resistant strains, such as *S. aureus* MRSA ATCC and patient isolates, compared to standardized strains. The most pronounced activity was observed for compound **1**, which achieved nearly complete inhibition (~99%) at c1 in the MRSA strain. This increased efficacy can be attributed to improved solubility and membrane permeability, provided by the MMEA moiety. In contrast, compound **2** exhibited lower biofilm inhibition in the *S. aureus* patient isolate (~37% at c1), possibly due to the denser extracellular matrix typical of patient-derived biofilms, which may act as a physical barrier, limiting the diffusion of the compound. Additionally, the lower lipophilicity and possible steric hindrance of compound **2** may further restrict its diffusion through the dense biofilm matrix, reducing its ability to reach and disrupt bacterial cells embedded within the biofilm.

## 3. Discussion

The distinct structures of compounds **1** and **2** influence their antibacterial activity against *S. aureus*, *E. coli* ATCC, and *S. typhimurium* ATCC by modulating permeability through the bacterial cell wall, membrane interactions, and the ability to reach intracellular targets. Structural differences between Gram-positive and Gram-negative bacteria further affect these effects.

In Gram-positive bacteria, compound **1** showed higher antibacterial activity against the reference strain *S. aureus* compared to clinical isolates. The reduced activity in clinical strains can be attributed to resistance mechanisms such as: (i) modified membrane composition or increased density of peptidoglycan layer that limit drug uptake; (ii) activation of efflux pumps; (iii) enzymatic inactivation [31].

The HMMEA^+^ cation may engage in electrostatic interactions with the negatively charged membrane of the Gram-positive bacteria, particularly with teichoic acids and carboxylic groups of the peptidoglycan layer. These interactions can affect the surface charge, cell wall permeability, and the transmembrane electrochemical potential (Δψ) [32]. The 2Cl5NB^−^ anion may interact with cell wall components via hydrogen bonding or π–π stacking [33], contributing to cell surface destabilization. Because of its partially hydrophobic character, it may facilitate limited penetration through the porous peptidoglycan layer and come into transient contact with the cytoplasmic membrane.

Once inside the cell, the 2Cl5NB^−^ anion can be enzymatically reduced by bacterial nitroreductases, generating reactive oxygen/nitrogen species (ROS/RNS) that induce oxidative stress, damaging DNA, proteins, and lipids, and inhibiting bacterial replication and viability. These complementary possible interactions of these ions could explain the enhanced antibacterial activity of compound **1** against *S. aureus* ATCC.

In contrast, compound **2** is expected to act through a distinct mechanism due to the absence of an organic cation. The K^+^ ions can influence the ionic balance across the bacterial membrane, causing slight perturbations of Δψ. The absence of an organic cation capable of interacting directly with the cell wall, as in compound **1**, likely limits membrane permeabilization and restricts the intracellular access of 2Cl5NB^−^. This results in lower antibacterial activity but greater selectivity, particularly against MRSA strains. The selective activity against MRSA strains, comparable to that of Gn at the highest concentration suggests a possible alternative mechanism of action to conventional resistance pathways [34], potentially involving disruption of potassium homeostasis or altering bacterial membrane potential [4].

In Gram-negative bacteria, the limited antibacterial efficacy of the studied compounds can be attributed to the structural and physiological barriers specific to these microorganisms. The outer membrane, enriched in LPS, acts as a selectively permeable barrier that restricts the passive diffusion of polar or hydrophilic molecules into the cell [35]. Additionally, the presence of active efflux pump systems can reduce the intracellular accumulation of the compound, limiting its antibacterial efficacy [36]. Gram-negative bacteria also possess efficient defense mechanisms against oxidative stress, including catalases, superoxide dismutases, and glutathione-dependent enzymes [37]. Compared with compound **2**, the presence of the HMMEA^+^ cation in compound **1** may promote electrostatic interactions with the negatively charged LPS layer, limiting penetration into the cell via porin channels [38] and causing partial membrane perturbation of the outer membrane potential. Once internalized, the 2Cl5NB^−^ anion is expected to act predominantly through intracellular mechanisms, possibly after passive diffusion or porin-mediated uptake. Among the Gram-negative strains, *E. coli* ATCC showed antibacterial sensitivity, with inhibition zones comparable to those of Sxt at the highest concentration, suggesting more efficient uptake through porins and a less restrictive LPS structure [39]. In contrast, *S. typhimurium* ATCC exhibited reduced activity, likely due to lower outer membrane permeability and increased efflux pump activity [40].

The mechanistic interpretations proposed here are based on structural features of the compounds and the known behavior of related molecules. Future studies will employ membrane integrity assays and microscopy techniques to elucidate the molecular basis of the observed antibacterial effects.

## 4. Materials and Methods

### 4.1. Synthesis and Structural Characterization Data

#### 4.1.1. Chemicals

The reagents (2Cl5NBH, MMEA, KI) and solvents (acetone and ethanol) used for the synthesis and crystallization were purchased from Signa-Aldrich (Milwaukee, WI, USA) and Merck (Darmstadt, Germany), respectively. All reagents were of analytical grade and used without additional purification.

#### 4.1.2. Synthesis of Compound **1**

Compound **1** was obtained by slowly adding a 15 mL acetone solution of MMEA (0.4 mL, 4.96 mmol) to a 15 mL acetone solution of 2Cl5NBH (1 g, 4.96 mmol). The clear, pale-yellow solution was stirred at RT for 1 h and subsequently allowed to evaporate slowly under ambient conditions. After several days, yellow crystals were formed, with an excellent yield (η ≈ 94%). The crystals were collected by vacuum filtration, washed with a small volume of acetone at RT and air-dried.

#### 4.1.3. Synthesis of Compound **2**

Compound **2** was synthesized by reacting equimolar quantities of compound **1** (0.15 mmol) with potassium iodide (0.15 mmol) in a 6 mL EtOH:H_2_O (1:1, *v*/*v*) mixture. The reaction mixture was first heated to 120 °C and then refluxed at 50 °C for 1 h. The resulting clear solution was filtered and left to cool gradually to RT. After approximately two months, colourless single crystals were obtained. (η ≈ 65%). The crystals were collected by vacuum filtration, washed with a solvent mixture, and air-dried.

#### 4.1.4. Polarized Optical Microscopy (POM) with Linkam Hot Stage

The melting and thermal degradation behavior of the compounds were examined using an Olympus BX53M polarized optical microscope (Olympus Corporation, Tokyo, Japan) equipped with a Linkam heating stage (Linkam Scientific Instruments, Tadworth, UK) and a 20× objective lens. Samples were heated at a rate of 10 °C min^−1^ under controlled conditions.

#### 4.1.5. Infrared Spectroscopy (IR)

The IR spectra were acquired using an ATR (Attenuated Total Reflectance) technique within the range of 650–4000 cm^−1^ on a PerkinElmer Spectrum-100 FTIR spectrometer (PerkinElmer Inc., Waltham, MA, USA).

#### 4.1.6. Single Crystal X-Ray Diffraction (SCXRD)

Suitable single crystals of compounds **1** and **2** were selected for X-ray diffraction measurements. SCXRD data were collected at room temperature using an Xcalibur E diffractometer equipped with a CCD area detector and a graphite monochromator, with a MoK radiation source. Unit cell parameters were determined, and all experimental datasets processed using CrysAlis software CrysAlisPro software (Oxford Diffraction Ltd., Version 1.171.37.35) [41]. The structures were solved using direct methods, with the SHELXS97 and SHELXL2014 programs [42,43] employed for structure solution and complete refinement of the proposed models. All non-hydrogen atoms were refined anisotropically. Hydrogen atoms bonded to carbon were placed geometrically and treated as riding atoms, whereas hydrogen atoms in water molecules were calculated based on hydrogen bonding interactions. Molecular drawings were generated using the MERCURY software (Version 2025.1.1) [44].

#### 4.1.7. Hirshfeld Surface Analysis

The Hirshfeld surfaces, along with their related 2D fingerprint plots, were investigated using CrystalExplorer software (Version 17.5) [45]. The calculations were performed based on the crystallographic information files (CIFs). Graphical plot surfaces have been mapped, with the normalized contact distance (*d*_norm_) ranging from −0.5278 to 1.1721 Å for compound **1** and −0.6121 to 1.2310 Å for compound **2**. The resulting maps show the intermolecular interactions in crystal structures, where red spots reflect dominant strong interactions, while white spots correspond to weaker interactions.

### 4.2. Assessment of Antibacterial Activity

#### 4.2.1. Microbial Strains

The antimicrobial activity was investigated against standardized and patient-isolated strains of Gram-positive (*S. aureus* ATCC 25923, *S. aureus* isolated from a hospitalized patient, MRSA ATCC 43300, MRSA isolated from a hospitalized patient) and Gram-negative bacteria (*S. typhimurium* ATCC 14028 and *E. coli* ATCC 25922). Five different concentrations (c1—100 µM; c2—50 µM; c3—25 µM; c4—10 µM; c5—1 µM) were prepared in PBS for each compound.

#### 4.2.2. Inhibition Zone Measurement

Antibacterial activity was evaluated using the disc diffusion test, which included both control antibiotics and the tested compounds against selected bacteria (Figure S2) [46,47]. Each microbial culture was initially diluted in sterile 0.9% NaCl solution to reach a turbidity equivalent to the 0.5 McFarland standard. The resulting suspensions were further diluted 1:10 and inoculated onto CHROM agar medium for bacteria (Oxoid Ltd. Basingstoke, Hampshire, UK), followed by uniform spreading on sterile Petri dishes. Sterile paper discs were placed on the agar surface, and 10 µL of each test compound was added to individual discs. Commercial antibiotic discs served as positive controls. The plates were incubated at 37° C for 24 h, after which the diameters of the inhibition zones were measured and compared across the tested compounds, Gn, and Sxt. Gn and Sxt were used as reference antibiotics for Gram-positive and Gram-negative bacteria, respectively. Inhibition zones ranged from 16–28 mm for Gn and 17–17.5 mm for Sxt. All tests were performed in triplicate and the results were expressed as the mean ± standard deviation (SD).

#### 4.2.3. Bacterial Cell Viability Evaluation

Bacterial cell viability was assessed by determining the respiratory activity of microbial cells using a colorimetric assay based on the conversion of 2,3,5-triphenyltetrazolium chloride (TTC) to formazan (Figure S3) [48,49]. Tetrazolium salts are reduced by the dehydrogenase enzyme, generating a red formazan product, which serves as an indicator of the total metabolic activity of viable cells [50]. Bacterial suspensions were prepared in tryptic soy broth (TSB) to match a turbidity of 0.5 McFarland standard. In each well of a sterile 96-well microtiter plate, 100 µL of bacterial inoculum was mixed with 50 µL of each tested compound at five different concentrations (c1–c5). Gn and Sxt were used as reference antibiotics for Gram-positive and Gram-negative bacteria, respectively. The plates were incubated at 37 °C for 24 h under continuous shaking at 120 rpm. After incubation, 50 µL of 0.5% TTC solution was added to each well, followed by a 2 h incubation at 37 °C. The conversion of TTC into red formazan was quantified spectrophotometrically at 460 nm using a BioTek Synergy/H1 microplate reader (BioTek Instruments, Winooski, VT, USA).

The inhibition rate was calculated using the following formula:

inhibition rate (%) = [(OD control − OD samples)/OD control] × 100(1)

where OD sample—the absorbance of bacteria treated with tested compounds (c1–c5); and OD control—the absorbance of the untreated bacterial cultures. All experiments were performed in triplicate and the results were expressed as the mean ± SD.

#### 4.2.4. Biofilm Inhibitory Activity Assessment

The inhibitory effect of the tested compounds on biofilm formation was evaluated using a modified version of the method described by Knežević and Petrović (2008) (Figure S4) [51]. The bacterial inoculum was prepared in TSB and adjusted to a turbidity equivalent to 0.5 McFarland standard. In each well of a sterile 96-well microplate, 100 µL of bacterial inoculum was added, followed by 50 µL of each tested compound at five different concentrations (c1–c5). The plates were incubated at 37 °C for 24 h. After incubation, the wells were washed twice with sterile 0.9% NaCl solution to remove non-adherent bacteria and then dried at 37 °C. Subsequently, 200 μL of 0.4% (*w*/*v*) crystal violet solution was added to each well and the plates were incubated for 20 min at 37 °C. Excess dye was removed by washing with distilled water, and the bound stain was solubilized with 200 µL of 30% (*v*/*v*) acetic acid for 30 min. The absorbance was measured at 570 nm using a BioTek Synergy/H1 microplate reader.

The biofilm formation inhibition rate was calculated using the following formula:

biofilm inhibition rate (%) = (OD sample/OD control) × 100(2)

where OD sample—the absorbance of biofilms formed in the presence of tested compounds (c1–c5); and OD control—the absorbance of the untreated biofilms. All experiments were conducted in triplicate and data were expressed as mean ± SD.

## 5. Conclusions

Two new 2Cl5NBH derivatives (compounds **1** and **2**) were synthesized and structurally characterized by single-crystal X-ray diffraction, IR spectroscopy, and Hirshfeld surface analysis, revealing distinct supramolecular architectures stabilized by hydrogen bonding, π–π stacking, and, in the case of compound **2**, additional metal–ligand and halogen interactions. The solid-state structures provide insights into the molecular composition and intermolecular interactions that define key physicochemical properties influencing biological responses under assay conditions.

Antibacterial screening revealed that compound **1** exhibited the highest activity, producing inhibition zones of up to 27 mm against *S. aureus* ATCC and 17 mm against *E. coli*, values comparable to those of Gn (28 mm) and Sxt (17 mm), respectively. Compound **2** showed selective efficacy against MRSA patient isolates, with inhibition zones of 16–14 mm across the tested concentrations, similar to Gn (16 mm). The results indicate that compound **1** exhibits broad-spectrum antibacterial and antibiofilm activity against both Gram-positive and Gram-negative bacteria. In contrast, compound **2** shows a more selective antibacterial profile, with pronounced efficacy against MRSA isolates.

## Figures and Tables

**Table 1:** Crystallographic and refinement data of compounds **1** and **2**.

	1	2
CCDC number	2480318	2480317
Formula	C_10_H_13_ClN_2_O_5_	C_7_H_5_ClKNO_5_
Formula weight	276.67	257.67
Crystal system	Triclinic	Orthorhombic
Space group	*P*-1	*P*2_1_2_1_2_1_
Z	2	4
*a* (Å)	7.2496 (10)	4.0939 (3)
*b* (Å)	7.9607 (15)	8.5779 (7)
*c* (Å)	12.3583 (14)	27.559 (2)
*α* (°)	102.928 (12)	90
*β* (°)	96.824 (10)	90
*γ* (°)	113.819 (15)	90
*V* (Å^3^)	618.19 (17)	967.77 (13)
*D*_c_ (g/cm^−3^)	1.486	1.768
*Μ* (mm^−1^)	0.324	0.824
*F* (000)	288	520
Crystal size (mm^3^)	0.80 × 0.30 × 0.18	0.60 × 0.04 × 0.03
Reflections collected/unique	3325/2167 [R(int) = 0.0198]	2380/1673 [R(int) = 0.0268]
Completeness (%)	99.5	99.5
Reflections with [*I* > 2σ(*I*)]	1581	1440
Parameters	165	143
GOF on *F*^2^	0.999	1.007
*R*_1_, w*R*_2_ [*I* > 2σ(*I*)]	0.0469, 0.1218	0.0556, 0.1369
*R*_1_, w*R*_2_ (all data)	0.0698, 0.1384	0.0649, 0.1446

## Data Availability

The data supporting the findings of this study are available within the article and its Supplementary Materials.

## Supplementary Materials

The following supporting information can be downloaded at: https://www.mdpi.com/article/10.3390/ijms262311607/s1.

## Abbreviations

The following abbreviations are used in this manuscript:

EWGs	Electron-withdrawing groups
ROS	Reactive oxygen species
RNS	Reactive nitrogen species
DNA	Deoxyribonucleic acid
LPS	Lipopolysaccharides
2Cl5NBH	2-chloro-5-nitrobenzoic acid
MRSA	Methicillin-resistant *Staphylococcus aureus*
SCXRD	Single-crystal X-ray diffraction
MMEA	Methylethanolamine
ATCC	American type culture collection
Δψ	Electrochemical potential
ATR	Attenuated Total Reflectance
CCD	Charge coupled device
MoK	Molybdenum K-alpha
CIFs	Crystallographic information files
PBS	Phosphate-buffered saline
Gn	Gentamicin
Sxt	Sulfamethoxazole -trimethoprim
SD	Standard deviation
TTC	2,3,5-triphenyltetrazolium chloride

## References

[1] Ahmed S.K., Hussein S., Qurbani K., Ibrahim R.H., Fareeq A., Mahmood K.A., Mohamed M.G. (2024). Antimicrobial resistance: Impacts, challenges, and future prospects. J. Med. Surg. Public Health.

[2] Ferraz M.P. (2024). Antimicrobial Resistance: The Impact from and on society according to one health approach. Societies.

[3] Oliveira M., Antunes W., Mota S., Madureira-Carvalho Á., Dinis-Oliveira R.J., Dias da Silva D. (2024). An overview of the recent advances in antimicrobial resistance. Microorganisms.

[4] Vestergaard M., Frees D., Ingmer H. (2019). Antibiotic resistance and the MRSA problem. Microbiol. Spectr..

[5] Douglas E.J.A., Wulandari S., Lovell S.D., Laabei M. (2023). Novel antimicrobial strategies to treat multi-drug resistant Staphylococcus aureus infections. Microb. Biotechnol..

[6] Zhou K., Sun L., Zhang X., Xu X., Mi K., Ma W., Zhang L., Huang L. (2023). *Salmonella* antimicrobials inherited and the non-inherited resistance: Mechanisms and alternative therapeutic strategies. Front. Microbiol..

[7] Synowiec A., Żyła K., Gniewosz M., Kieliszek M. (2021). An effect of positional isomerism of benzoic acid derivatives on anti-bacterial activity against *Escherichia coli*. Open Life Sci..

[8] Crisan M., Halip L., Bourosh P., Chicu S.A., Chumakov Y. (2017). Synthesis, structure and toxicity evaluation of ethanolamine nitro/chloronitrobenzoates: A combined experimental and theoretical study. Chem. Cent. J..

[9] Cruz Romero M.C., Murphy T., Morris M., Cummins E., Kerry J.P. (2013). Antimicrobial activity of chitosan, organic acids and nano-sized solubilisates for potential use in smart antimicrobially-active packaging for potential food applications. Food Control.

[10] Sullivan D.J., Azlin-Hasim S., Cruz-Romero M., Cummins E., Kerry J.P., Morris M.A. (2020). Antimicrobial effect of benzoic and sorbic acid salts and nano-solubilisates against *Staphylococcus aureus*, *Pseudomonas fluorescens* and chicken microbiota biofilms. Food Control.

[11] Li Z., Lin H., Zhou J., Chen L., Pan Z., Fang B. (2021). Synthesis and antimicrobial activity of the hybrid molecules between amoxicillin and derivatives of benzoic acid. Drug Dev. Res..

[12] Siddiqui W.A., Khalid M., Ashraf A., Shafiq I., Parvez M., Imran M., Irfan A., Hanif M., Khan M.U., Sher F. (2022). Antibacterial metal complexes of o--sulfamoylbenzoic acid: Synthesis, characterization, and DFT study. Appl. Organomet. Chem..

[13] Faleye O.S., Boya B.R., Lee J.H., Choi I., Lee J. (2023). Halogenated antimicrobial agents to combat drug-resistant pathogens. Pharmacol. Rev..

[14] Chiodi D., Ishihara Y. (2023). “Magic Chloro”: Profound effects of the chlorine atom in drug discovery. J. Med. Chem..

[15] Li J., Song S., Huang W., Fan H., Zhou Z. (2023). Novel drug-drug salts of enoxacin with enhanced antibacterial activity: Insights from solubility and lipid-water partition coefficient. J. Mol. Liq..

[16] Croitor L., Vlase G., Vlase T., Bourosh P.N., Chumakov Y.M., Crisan M. (2022). Relationship between crystal structure and thermal properties of polymorphic system methylethanolammonium 2-chloro-4-nitrobenzoate. J. Therm. Anal. Calorim..

[17] Crisan M., Vlase G., Szerb E.I., Vlase T. (2018). Thermal and kinetics studies of primary, secondary and tertiary alkanolammonium salts of 4-nitrobenzoic acid. J. Therm. Anal. Calorim..

[18] Crisan M., Petric M., Vlase G., Vlase T., Siminel A.V., Bourosh P., Croitor L. (2022). Organic salt versus salt cocrystal: Thermal behavior, structural and photoluminescence investigations. J. Therm. Anal. Calorim..

[19] Chicu S.A., Grozav M., Kurunczi L., Crisan M. (2008). SAR for amine salts of carboxylic acids to *Hydractinia echinata*. Rev. Chim..

[20] Patel D., Witt S.N. (2017). Ethanolamine and phosphatidylethanolamine: Partners in health and disease. Oxid. Med. Cell. Longev..

[21] Pandey A., Boros E. (2021). Coordination complexes to combat bacterial infections: Recent developments, current directions and future opportunities. Chemistry.

[22] Danilescu O., Bourosh P., Bulhac I., Shova S., Kravtsov V.C., Caraba M.N., Caraba I.V., Popescu R., Crisan M., Haidu D. (2024). Laminated dihydrazone Zn(II) coordination polymer with prospects for sensory and multifunctional biomedical applications. Polyhedron.

[23] Arunadevi A., Porkodi J., Ramgeetha L., Raman N. (2019). Biological evaluation, molecular docking and DNA interaction studies of coordination compounds gleaned from a pyrazolone incorporated ligand. Nucleosides Nucleotides Nucleic Acids.

[24] Bharti S.K., Singh S.K. (2009). Metal-based drugs: Current use and future potential. Der. Pharm. Lett..

[25] Stautz J., Hellmich Y., Fuss M.F., Silberberg J.M., Devlin J.R., Stockbridge R.B., Hänelt I. (2021). Molecular mechanisms for bacterial potassium homeostasis. J. Mol. Biol..

[26] Gorobet A., Crisan M.E., Bourosh P., Siminel A.V., Croitor L. (2021). Supramolecular architectures and photoluminescent properties of triethanolammonium 4-nitrobenzoate salt and its Ni(II) complexes. Polyhedron.

[27] Gorobet A., Crisan M.E., Bourosh P., Croitor L. (2021). Structural investigation and Hirshfeld surface analysis of Cu(II) triethanolamine 4-nitrobenzoate. Rev. Roum. Chim..

[28] Gorobet A., Crisan M., Petric M., Bourosh P., Croitor L. (2018). Structural study of Ca(II) coordination compound with triethanolamine and 4-nitrobenzoic acid. Rev. Roum. Chim..

[29] Pavia D.L., Lampman G.M., Kriz G.S., Vyvyan J.R. (2015). Introduction to Spectroscopy.

[30] Mohan J. (2004). Organic Spectroscopy: Principles and Applications.

[31] Abebe A.A., Birhanu A.G. (2023). Methicillin-Resistant *Staphylococcus aureus*: Molecular mechanisms underlying drug resistance development and novel strategies to combat. Infect. Drug Resist..

[32] Bennett W.F.D., Fox S.J., Sun D., Maupin C.M. (2022). bacterial membranes are more perturbed by the asymmetric versus symmetric loading of amphiphilic molecules. Membranes.

[33] Wheeler S.E. (2013). Understanding substituent effects in noncovalent interactions involving aromatic rings. Acc. Chem. Res..

[34] Akshay S.D., Deekshit V.K., Mohan Raj J., Maiti B. (2023). Outer membrane proteins and efflux pumps mediated multi-drug resistance in *Salmonella*: Rising threat to antimicrobial therapy. ACS Infect. Dis..

[35] Delcour A.H. (2009). Outer membrane permeability and antibiotic resistance. Biochim. Biophys. Acta.

[36] Kumawat M., Nabi B., Daswani M., Viquar I., Pal N., Sharma P., Tiwari S., Sarma D.K., Shubham S., Kumar M. (2023). Role of bacterial efflux pump proteins in antibiotic resistance across microbial species. Microb. Pathog..

[37] Brand C., Newton-Foot M., Grobbelaar M., Whitelaw A. (2025). Antibiotic-induced stress responses in Gram-negative bacteria and their role in antibiotic resistance. J. Antimicrob. Chemother..

[38] Choi U., Lee C.R. (2019). Distinct roles of outer membrane porins in antibiotic resistance and membrane integrity in *Escherichia coli*. Front. Microbiol..

[39] Snyder D.S., McIntosh T.J. (2000). The lipopolysaccharide barrier: Correlation of antibiotic susceptibility with antibiotic permeability and fluorescent probe binding kinetics. Biochemistry.

[40] Mahendran K.R., Kreir M., Weingart H., Fertig N., Winterhalter M. (2010). Permeation of antibiotics through *Escherichia coli* OmpF and OmpC porins: Screening for influx on a single-molecule level. J. Biomol. Screen..

[41] (2003). CrysAlis RED.

[42] Sheldrick G.M. (2008). A short history of SHELX. Acta Crystallogr. A.

[43] Sheldrick G.M. (2015). Crystal structure refinement with SHELXL. Acta Crystallogr. C.

[44] Macrae C.F., Edgington P.R., McCabe P., Pidcock E., Shields G.P., Taylor R., Towler M., van de Streek J. (2006). Mercury: Visualization and analysis of crystal structures. J. Appl. Crystallogr..

[45] Spackman P.R., Turner M.J., McKinnon J.J., Wolff S.K., Grimwood D.J., Jayatilaka D., Spackman M.A. (2021). *CrystalExplorer*: A program for Hirshfeld surface analysis, visualization and quantitative analysis of molecular crystals. J. Appl. Crystallogr..

[46] Clinical and Laboratory Standards Institute (CLSI) (2017). Performance Standards for Antimicrobial Susceptibility Testing.

[47] Filimon M.N., Popescu R., Sinitean A., Maniu P., Dumitrescu G., Verdes D., Vlad C.S. (2018). The Assessment of chitosan solutions effects on bacterial strains. Rev. Chim..

[48] Vlad C.S., Vlaia L., Vlaia V., Dumitraşcu D., Filimon M.N., Popescu R., Cimporescu A., Dehelean C., Onwubiko C.E., Daliborca C.V. (2019). Chromatographic analysis and antibacterial potential of extracts of *Gnetum africanum*. Farmacia.

[49] Popescu R., Filimon M.N., Vlad D.C., Verdes D., Moatar A., Moisei G., Gurani K., Caraba I.V., Petculescu Ciochina L., Pinzaru I. (2021). Antiproliferative and antibacterial potential of tetrahexylammonium bromide-based ionic liquids. Exp. Ther. Med..

[50] Berridge M.V., Herst P.M., Tan A.S. (2005). Tetrazolium dyes as tools in cell biology: New insights into their cellular reduction. Biotechnology Annual Review.

[51] Knezevic P., Petrovic O. (2008). A colorimetric microtiter plate method for assessment of phage effect on Pseudomonas aeruginosa biofilm. J. Microbiol. Methods.

