# Supplementary material for: From Structure to Function: 2-Chloro-5-nitrobenzoic Acid Derivatives as Potential Next-Generation Antibacterials
Source: Int J Mol Sci. 2025 Nov 29;26(23):11607. doi: 10.3390/ijms262311607 (PMC12692062; doi:10.3390/ijms262311607)
Supplement: Supplementary file 1 [file ijms-26-11607-s001.zip › ijms-3967694-supplementary.pdf]

Supplementary materials

From Structure to Function: 2-Chloro-5-nitrobenzoic Acid Derivatives as Potential Next-Generation Antibacterials

Lilia Croitor [1,†], Anastasia Gorobet [1], Marioara Nicoleta Caraba [2,3,†], Pavlina Bourosh [1], Ion Valeriu Caraba [3,4], Daniela Haidu [5] and Manuela Crisan [5,*]

[1] Institute of Applied Physics, Moldova State University, 5 Academiei Str., MD2028 Chisinau, Moldova; lilia@gmail.com (L.C.); nasteagorobet@gmail.com (A.G.); bourosh.xray@gmail.com (P.B.)
[2] Department of Cellular and Molecular Biology, Faculty of Medicine, "Victor Babeș" University of Medicine and Pharmacy Timisoara, 300041 Timisoara, Romania; nicoleta.caraba@umft.ro
[3] ANAPATMOL Research Center, "Victor Babeș" University of Medicine and Pharmacy Timisoara,
300041 Timisoara, Romania; caraba_i@animalsci-tm.ro
[4] Faculty of Bioengineering of Animal Resources, University of Life Sciences "King Mihai I" from Timisoara, 119 Calea Aradului, 300645 Timisoara, Romania
[5] "Coriolan Dragulescu" Institute of Chemistry, Romanian Academy, 24 Mihai Viteazu Bd.,
300223 Timisoara, Romania; dana_141@yahoo.com or danielahaidu1@gmail.com
* Correspondence: mcrisan@acad-icht.tm.edu.ro
† These authors contributed equally to this work.

2. Results

2.1 Synthesis and structural characterization

Table S1. Selected geometric parameters of hydrogen bonds (Å, ◦) in compound **1**.

D-H...A	d(D-H)	d(H...A)	d(D...A)	<(DHA)	Symmetry transformations for acceptor
O(5)-H(5A)...O(2)	0.82	1.83	2.648(3)	176.9	x+1,y,z+1
N(2)-H(2A)...O(1)	0.89	1.87	2.721(3)	159.0	x+1,y,z+1
N(2)-H(2B)...O(5)	0.89	2.03	2.827(3)	149.2	-x+1,-y,-z+2

Table S2. Bond distances (Å) and bond angles (◦) in the metal coordination cores of compound **2.**

K(1)-O(1W)	2.726(6)	K(1)-O(3)#3	2.881(5)
K(1)-O(1)#1	2.761(6)	K(1)-O(2)#2	3.321(5)
K(1)-O(1)	2.770(6)	K(1)-Cl(1)	3.523(3)
K(1)-O(1W)#1	2.801(6)	K(1)-Cl(1)#1	3.583(3)
K(1)-O(1)#2	2.850(4)		
O(1W)-K(1)-O(1)#1	169.54(13)	O(1)#1-K(1)-O(3)#3	119.36(19)
O(1W)-K(1)-O(1)	84.13(16)	O(1)-K(1)-O(3)#3	108.02(17)
O(1)#1-K(1)-O(1)	95.48(14)	O(1W)#1-K(1)-O(3)#3	81.45(17)
O(1W)-K(1)-O(1W)#1	95.59(15)	O(1)#2-K(1)-O(3)#3	151.67(18)
O(1)#1-K(1)-O(1W)#1	82.91(16)	O(1W)-K(1)-O(2)#2	69.66(14)
O(1)-K(1)-O(1W)#1	169.69(13)	O(1)#1-K(1)-O(2)#2	101.67(15)
O(1W)-K(1)-O(1)#2	85.74(16)	O(1)-K(1)-O(2)#2	118.54(13)
O(1)#1-K(1)-O(1)#2	83.83(14)	O(1W)#1-K(1)-O(2)#2	52.31(13)

O(1)-K(1)-O(1)#2	83.66(14)	O(1)#2-K(1)-O(2)#2	41.49(13)
O(1W)#1-K(1)-O(1)#2	86.03(16)	O(3)#3-K(1)-O(2)#2	113.05(16)
O(1W)-K(1)-O(3)#3	70.42(18)		

Symmetry transformations used to generate equivalent atoms: #1 x+1,y,z #2 x+1/2,-y+1/2,-z+2 #3 x+1,y+1,z

Table S3. Band assignments (cm^{-1}) of vibrational modes observed in ATR-FTIR spectra of tested compounds

	$\nu_{as}COO^-$	ν_sCOO^-	$\nu C{=}O$	$\nu C\text{-}O$	$\nu C\text{-}N$	νNH_2^+	δNH_2^+	$\nu O\text{-}H$	$\nu C\text{-}H$ arom	$\nu_{as}NO_2$	ν_sNO_2	$\nu(C\text{-}Cl)$
**2Cl5NBH**	-	-	1685	1250	-	-	-	-	3105	1528	1346	727
**1**	1584	1403	-	-	1076	bands 3100-2300	1584	-	3104	1516	1347	733
**2**	1596	1401	-	-	-	-	-	3438, 3219	3104	1521	1340	742

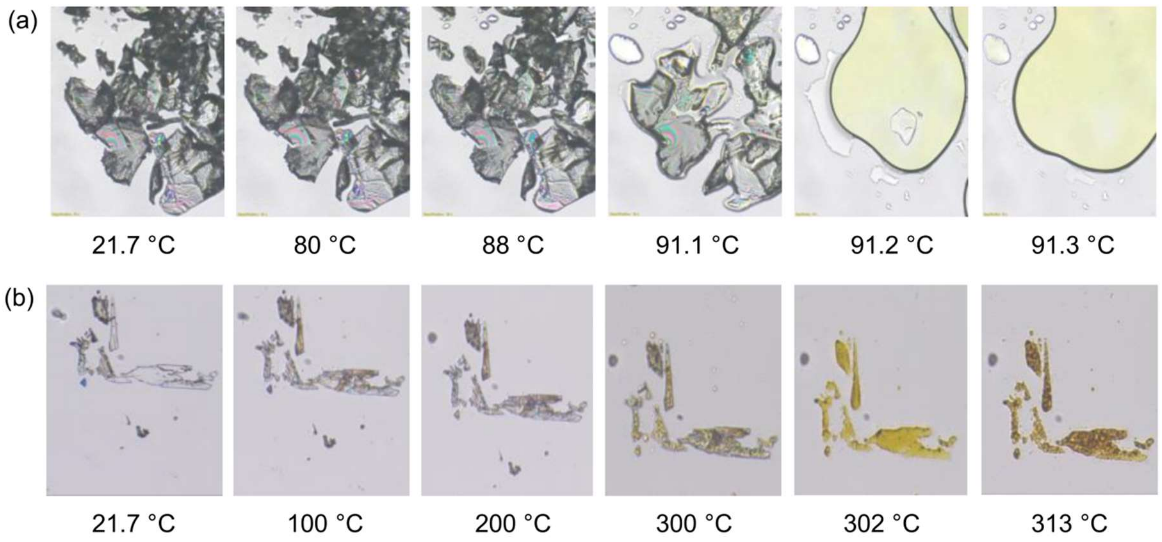

Figure S1. Optical micrographs of compounds **1** and **2** showing melting (a) and thermal degradation (b), respectively

2.2 Computational study

Table S4. Intermolecular interactions for compounds **1** and **2** derived from Hirshfeld surface analysis (*d_norm* surfaces)

Interaction category	Contact Types Included	Compound 1 (%)	Compound 2 (%)
**Hydrogen bonding**	O-H	37.4	30
	N-H	0.2	0.9
	Cl-H	13.3	4.3
	K-H	0	2.3
	total	**50.90%**	**38%**
**van der Waals + π**	H-H	26.8	12.8
	C-H	9.2	4.1
	C-C	2.8	6.7
	C-N	1.3	1.3
	O-C	3.3	3.7

	O-O	1.2	2.1
	total	**44.60%**	**30.70%**
**Halogen contacts**	Cl-Cl	0	0.5
	Cl-O	3	9
	Cl-N	0	0.1
	Cl-C	0	2.7
	total	**3.00%**	**12.30%**
**Metal coordination contacts**	K-Cl	0	1.8
	K-O	0	15.9
	K-C	0	0.7
	total	**0**	**18.40%**

All percentage values correspond to distinct interaction types; overlapping or duplicate contacts that could contribute to multiple categories have been omitted to ensure clear and independent representation of each interaction.

4. Materials and Methods

4.2 Assessment of antibacterial activity

4.2.2 Inhibition zone measurement

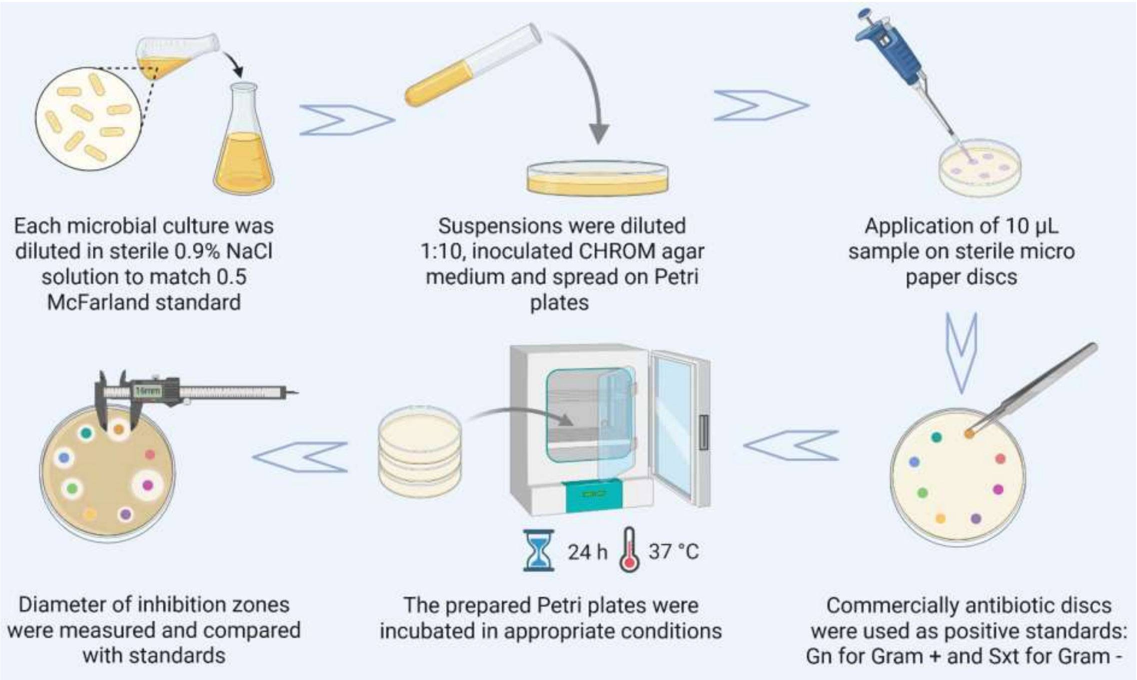

Figure S2. Main steps involved in the disk diffusion test for antibacterial activity evaluation

4.2.3 Bacterial cell viability evaluation

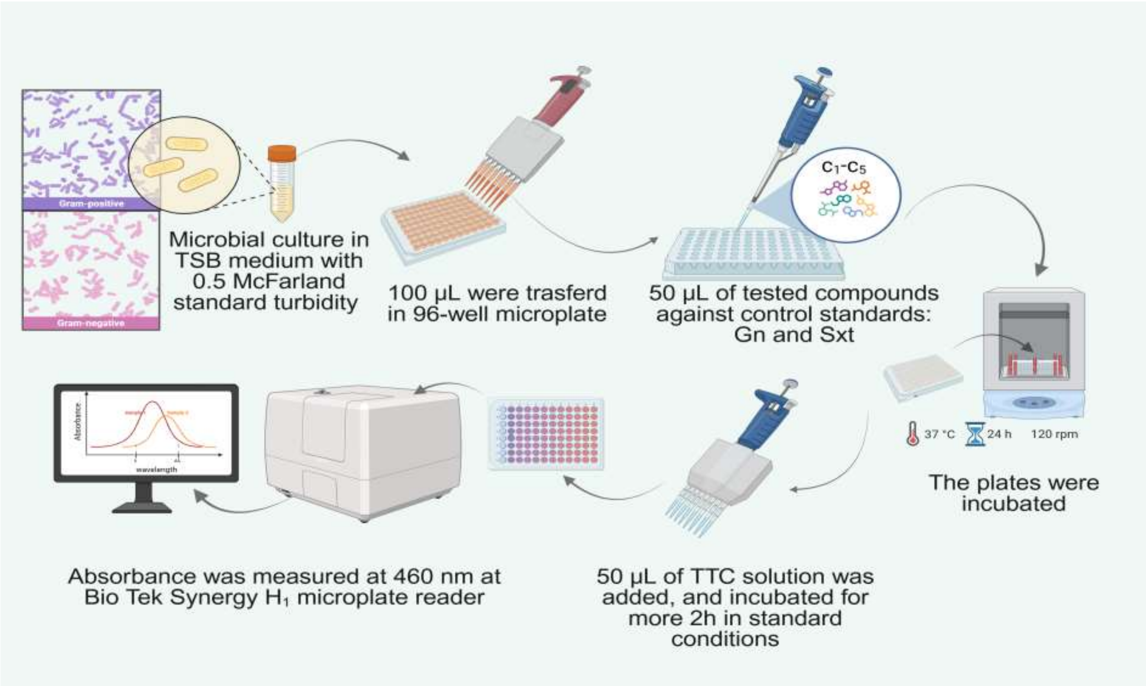

Figure S3. Sequential steps involved in the bacterial cell viability test

4.2.4 Biofilm inhibitory activity assessment

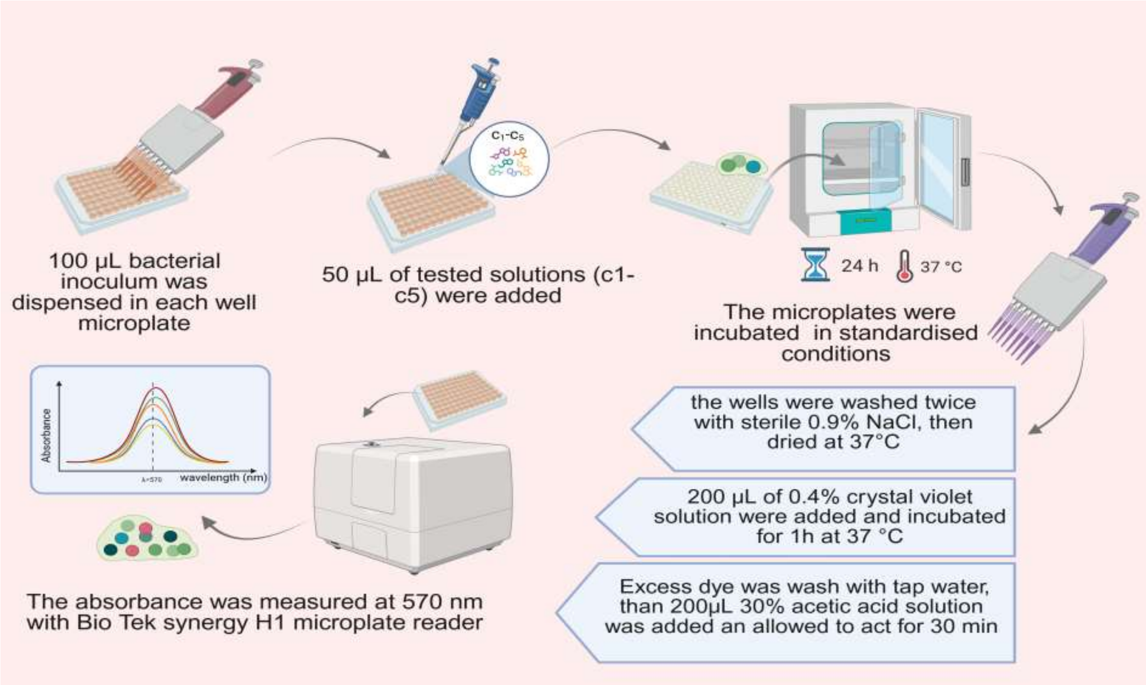

Figure S4. Experimental steps for evaluating the biofilm inhibitory activity of tested compounds
